# MP-Stain-Detector: A Learning-Based Stain Detection Method with a Multispectral Polarization Optical System

**DOI:** 10.3390/s26051703

**Published:** 2026-03-08

**Authors:** Shun Zou, Pei An, Xiaoming Liu, Zuyuan Zhu, Yan Song, Tao Song, You Yang

**Affiliations:** 1 School of Electronic Information and Communications, Huazhong University of Science and Technology, Wuhan 430074, China; 2 Wuhan National Laboratory for Optoelectronics, Wuhan 430074, China; 3 Hubei Optics Valley Laboratory, Wuhan 430074, China; 4 Qingdao Haier Intelligent Technology R&D Co., Qingdao 266103, China

**Keywords:** stain detection, multispectral polarization imaging, stain classification, the robotic sweepers

## Abstract

Stain detection is crucial for robotic sweepers, enabling them to assess environmental hygiene and execute precise cleaning tasks. However, in complex indoor scenarios, highly accurate stain detection remains a significant challenge, as the visual features of stains are often obscured by ambient light, background textures, and specular reflections. Most existing deep learning methods rely predominantly on standard Red-Green-Blue (RGB) images, which lack sufficient discriminative features to robustly distinguish stains from complex backgrounds or accurately classify diverse contaminants. To address these limitations, we propose a deep learning stain detection framework integrated with a multispectral polarization optical system. First, to extract discriminative optical features, we design a lightweight multispectral polarization optical module tailored for integration into robotic sweepers. It captures rich spectral and polarization features while effectively suppressing specular reflections. Second, to enhance feature representation capabilities, we develop a multispectral polarization (MP)-based stain detector, named MP-stain-detector, which fuses spectral composition data with polarization texture features. Third, to support rigorous model training and evaluation, we construct a comprehensive dataset, the MP-Stain-dataset, collected in real-world home scenarios. Experiments on the MP-Stain-dataset demonstrate that our method improves the overall mean accuracy by 2.44%, and by 5.72% for the challenging light-colored liquid category compared to conventional approaches.

## 1. Introduction

Stain detection is a core perception capability for robotic sweepers. It serves as the foundation for autonomous operation, enabling the system to assess environmental hygiene and execute precise cleaning tasks. These devices identify contaminated areas and execute targeted cleaning strategies in an automated and efficient manner [1]. Recently, deep learning-based stain detection has emerged as a prominent technology [2,3]. It enables robotic sweepers to accurately recognize diverse contaminants and implement adaptive cleaning strategies, serving as a crucial component for their overall intelligence and efficiency [4]. However, accurate detection remains a significant challenge for mainstream methods based on RGB cameras in complex indoor scenarios [5]. The visual features of stains are often obscured by ambient light, background textures, and specular reflections. RGB images captured by standard optical cameras lack sufficiently discriminative features to differentiate between the material properties of stains and their backgrounds. Therefore, existing learning methods fail to robustly identify various stains on floors with complex patterns. To address these challenges, we propose a deep learning stain detection framework integrated with a multispectral polarization optical system. Given the limited feature representation of RGB images, we aim to leverage multidimensional optical information to enhance the discriminability of stain features. First, to extract discriminative optical features from various stains, we design a lightweight multispectral polarization optical module tailored for integration into robotic sweepers. Extensive stain saliency analysis demonstrates that this system captures rich spectral and polarization features while effectively suppressing specular reflections, a technique validated in other harsh environment detection tasks [6]. Second, we develop a multispectral polarization-based stain detector, named the MP-stain-detector. It fuses spectral composition data with polarization texture features to significantly enhance feature representation capabilities. Third, to effectively train the proposed model, we construct a comprehensive dataset, the MP-Stain-dataset, comprising real-world stains to support rigorous model training and evaluation. Experimental results on this dataset demonstrate that our method achieves superior performance in detection accuracy and classification precision compared to conventional approaches. The main contributions of this work are summarized as follows: (1) the development of a compact multispectral polarization imaging system tailored for robotic sweepers, which captures multidimensional optical information to effectively suppress specular reflections and enhance stain feature extraction; (2) the proposal of a novel multimodal detection network, the MP-stain-detector, that fuses spectral and polarization features to significantly improve detection accuracy and robustness; and (3) the construction of a comprehensive multispectral polarization stain dataset, the MP-Stain-dataset, collected in real-world scenarios to support the training and evaluation of the proposed method.

## 2. Related Works

Over the past two decades, research on stain detection for robotic sweepers has evolved significantly. Generally, these approaches can be categorized into two main streams: traditional detection methods and deep learning-based methods.

Traditional Detection Methods: Early research primarily relied on traditional computer vision techniques and specific optical sensors. These methods typically utilize handcrafted features extracted from RGB or Red-Green-Blue-Depth (RGB-D) data or leverage multispectral analysis to identify contaminants. Milinda et al. [7] achieved targeted cleaning by detecting and distinguishing mud from ordinary dirt on textured surfaces. Their approach differentiates common dirt from mud stains by combining maximum stable extremal regions (MSER) for particle localization, spectral residual filtering for anomaly highlighting, and RGB color filtering alongside variance thresholding. Ramalingam et al. [5] established a three-tier visual dirt detection framework, while Olorunfemi et al. [8] implemented solar panel dust detection using RGB correlation coefficients. Furthermore, Wu et al. [9] proposed a public restroom cleaning robot equipped with redundant dual arms, which employs a clamping spray gun and optimized path planning to effectively remove stubborn stains. Similarly, Rodríguez et al. [10] developed a laser cleaning robot system integrating a 6-DOF arm and 3D surface capture to automatically generate cleaning paths for complex 3D objects. In the field of new energy maintenance, Gao [11] designed an unmanned intelligent cleaning robot for distributed photovoltaic power plants. By optimizing the hardware layout to balance the center of gravity and employing path planning algorithms, the robot achieved significant improvements in obstacle traversal and cleaning adaptability. Although these methods established the groundwork, they rely heavily on handcrafted features and strict environmental assumptions. Vision-based approaches are highly sensitive to lighting variations and background textures, leading to poor robustness in complex, dynamic indoor scenarios. Traditional multispectral systems, while effective in enhancing contrast, often involve complex, bulky hardware and high costs, lacking the compactness required for robotic sweepers.
Deep Learning-Based Methods: With the advancement of artificial intelligence, deep learning architectures have dominated recent detection research. These approaches leverage neural networks to automatically extract hierarchical features, offering superior generalization in complex environments. Bormann et al. [3] enhanced YOLOv3 to develop a robust, rapid, and generalizable visual dirt and object detection system adaptable to various floor types in novel environments. Xu et al. [6] proposed an improved YOLOv8n framework for road target detection in harsh environments (e.g., rain, snow, and fog) by integrating space-to-depth convolution and polarized self-attention mechanisms to compensate for fine-grained information loss. Gao et al. [12] optimized the YOLOv8-seg [13] model with mRMR feature selection, utilizing the improved model to segment insulator regions. More recently, Li et al. [14] developed an improved YOLOv8-based framework combined with Deep OC-SORT for the dynamic detection and tracking of tobacco debris. Their work incorporates a Swin Transformer into the backbone and utilizes a Dynamic Head to achieve high precision and robustness in industrial production lines, demonstrating the effectiveness of task-specific architectural refinements. In the same domain, Huang et al. [15] utilized an FPGA-accelerated ResNet18 model to achieve real-time online recognition of cigarette filter defects, highlighting the importance of hardware-algorithm co-design. Canedo et al. [2] combined synthetic data augmentation with YOLOv5 [16], yet demonstrated inadequate performance for liquid stain detection. Addressing limited samples in a related field, Zeng et al. [17] proposed a ceramic rare defect amplification method based on TC-CycleGAN to alleviate the class imbalance problem. Similarly, in medical diagnostics, Gao et al. [18] utilized digital holography and deep learning to achieve virtual staining for colorectal cancer metastasis detection, demonstrating the potential of label-free imaging. In addition, Zhu et al. [19] proposed a multi-source data fusion network combining image and depth data to effectively suppress interference like stains and mineral lines for wood surface defect segmentation. Moreover, to address the limited depth of field in conventional imaging, Li et al. [20] proposed MVMFCam, a multi-view multi-focus computational imaging system that achieves all-in-focus imaging for dynamic scenes through a specialized fusion network. Furthermore, Li et al. [21] introduced DFS-Net, an aberration-aware network capable of synthesizing high-quality all-in-focus images and dense focal stacks from misaligned inputs. Similarly, to tackle the trade-off between high resolution and large field of view in aerial remote sensing, Zhang et al. [22] developed a light field-guided optical synthetic aperture system using master-slave UAVs. More recently, Sarah et al. [4] integrated RGB-D sensors, deep learning, and reconfigurable robots within an AI ecosystem, and Zhao et al. [23] proposed the MSHDSC segmentation model with MSFFM modules while releasing the first heliostat dirt dataset. Additionally, Wu et al. [24] addressed the issue of camera sensor contamination in autonomous vehicles by constructing a dedicated dirt dataset and utilizing YOLOv5 for effective recognition. Furthermore, the evolution of object detection frameworks has recently seen the integration of state-of-the-art architectures to handle complex environmental constraints. For instance, Long et al. [25] proposed IM-YOLO, an improved model based on the Mamba-YOLO architecture specifically for small object detection in aerial imagery. By incorporating a spatial-channel synergistic attention mechanism and a feature complementary fusion module, IM-YOLO effectively enhances the representation of shallow-layer features while suppressing background interference. This aligns with the necessity in stain detection to distinguish subtle target features from high-interference backgrounds, as demonstrated by the performance improvements on datasets like VisDrone2019. Although these methods exhibit improved generalization, they are often data-hungry and computationally intensive. More critically, relying solely on RGB or RGB-D information limits their ability to detect stains that lack distinct color contrast or texture features, such as transparent liquids or fine powders.
Discussion: Despite the advancements in stain detection, significant challenges persist in complex indoor environments. Traditional methods struggle with robustness under dynamic illumination and background variations, while current deep learning methods are fundamentally limited by the insufficient optical information captured by RGB sensors. Specifically, the lack of spectral information makes it difficult to distinguish stains from floor patterns with similar colors, while the inability to filter specular reflections often leads to edge blurring and feature loss, particularly for liquid contaminants. To address these challenges, we propose a deep learning stain detection framework integrated with a multispectral polarization optical system. Unlike traditional approaches, our method, the MP-stain-detector, captures richer spectral features to reveal material properties and utilizes polarization to effectively suppress background light and refractive interference, thereby fundamentally improving detection accuracy and stability. Furthermore, we construct a specialized acquisition system tailored for robotic sweepers to mitigate environmental interference at the data source and establish a comprehensive multispectral polarization dataset. Experimental results on the MP-Stain-dataset demonstrate that our method improves the overall mean accuracy by 2.44%, and by 5.72% for the challenging light-colored liquid category.

## 3. Methodology

### 3.1. Multispectral Polarization Optical System Design

**Figure 1:**
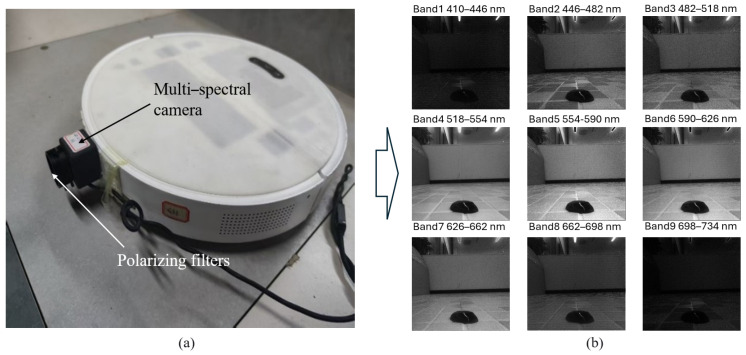
(**a**) A robotic vacuum cleaner equipped with a polarization-multispectral sensor data acquisition system. (**b**) Images captured across multiple spectral bands.

To address the limitations of RGB sensors in stain detection—specifically their inability to distinguish materials with similar colors and their susceptibility to specular reflections—we developed a specialized multispectral polarization optical acquisition system. The overall structure of this data acquisition device is illustrated in Figure 1a. The system is integrated into a standard robotic sweeper platform to ensure that data collection accurately reflects real-world cleaning scenarios.

The optical system comprises three key components: a multispectral imaging module, a polarization filtering module, and an adjustable illumination module. To clarify the detailed optical scheme along the optical axis: scattered and reflected light from the floor stains first propagates through the polarization filtering module mounted at the very front of the system. This pre-filtered light then enters the main imaging lens assembly. After passing through the lens, the focused light reaches the multispectral imaging module. Specifically, instead of using external beam splitters or rotating filter wheels, the CM020 multispectral camera utilizes a snapshot imaging architecture featuring nano-scale spectral filter arrays directly coated onto the underlying Complementary Metal-Oxide-Semiconductor (CMOS) pixel surface (on-chip filtering). Each pixel or pixel cluster is coated to receive only a specific wavelength band. This strict cascaded optical arrangement ensures that the incident light is first cleared of specular noise before being spectrally separated at the pixel level, allowing the sensor to capture optical information across a broad range of discrete spectral bands, extending from the visible to the near-infrared spectrum. As shown in Figure 1b, the system acquires images of the same stain across multiple spectral bands. This capability allows it to capture the unique spectral reflectance signatures of different stain materials, which are often indistinguishable in the standard RGB color space.

To mitigate the interference caused by specular reflections—which are common on liquid stains and smooth floor tiles—a polarization filtering module is mounted in front of the imaging lens, as depicted in the system exterior view in Figure 1a. By selectively filtering light vibrating in specific orientations (corresponding to projections of the electric field polarization vector, specifically sampled at 45°, 90°, and 135° during our data collection), this module effectively suppresses glare and highlights, thereby enhancing the visibility of stain textures and boundaries. These captured optical features provide a comprehensive representation of the physical properties of the stains. Furthermore, it is crucial to emphasize that the spectral and polarization images are acquired simultaneously rather than sequentially. Because the fixed polarization filter is integrated directly via an optical mount in front of the lens, and the multispectral separation occurs intrinsically at the sensor level through the nano-coated arrays, the raw images naturally possess both distinct spectral bands and specific polarization properties within a single exposure. This simultaneous acquisition mechanism is vital for moving robotic sweepers, as it completely eliminates spatial misalignment and motion artifacts.

The illumination module provides stable and adjustable lighting to simulate various indoor environments.

### 3.2. Stain Detector Based on Multispectral Polarization Images

**Figure 2:**
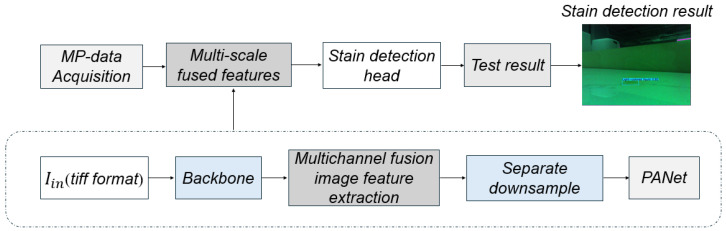
Multispectral polarization stain detection feature fusion method.

As illustrated in Figure 2, we design a specialized deep learning detector to fully leverage the rich optical information provided by multispectral polarization data for high-precision stain detection. The fused multispectral polarization images serve as the input to the detector, which then predicts both the precise locations and categories of the stains. The overall architecture consists of three main components: a highly efficient feature extraction backbone, a path aggregation network (PANet) for multi-scale feature fusion, and a decoupled detection head.

#### 3.2.1. Multispectral Polarization Input Construction

The input to the network is a composite tensor constructed from the captured multispectral and polarization data. A set of images across different bands. A total of N=10 distinct bands are selected to form the input. Specifically, nine spectral bands are utilized to construct a spectral feature cube $S\in R^{H\times W\times (N-1)}$. Additionally, the multispectral camera simultaneously captures a broadband grayscale image alongside the narrow spectral bands. We introduce an inverted version of this original grayscale image, denoted as $G_{inv}\in R^{H\times W\times 1}$ (calculated via a straightforward pixel-wise inversion, $255-I_{raw}$), to emphasize spatial structures. The rationale for this inversion is that stains typically absorb or scatter incident light, appearing darker than the clean, reflective background floor in the raw grayscale intensity map. By inverting the image, these anomalous dark regions are transformed into high-intensity features. Since convolutional neural networks generally exhibit stronger activations for higher pixel values, this inversion explicitly enhances the saliency of the stain textures and boundaries, effectively preserving and prioritizing crucial structural information in the original pixel space. These components are concatenated along the channel dimension to form the final network input tensor $I_{in}$:

(1) $$I_{in}=\mathrm{Concat}\left(S,G_{inv}\right)\in R^{H\times W\times N}$$

where *H* and *W* denote the height and width of the input image, respectively, and N=10 represents the total number of input channels. This input construction is crucial as it integrates two complementary optical modalities. The spectral bands provide material-specific reflectance signatures that are invariant to color changes, enabling the network to distinguish stains from background patterns based on their chemical composition. Simultaneously, the polarization channel, which utilizes raw polarization-resolved intensities to extract polarization texture features, enhances surface texture and suppresses specular reflections. This approach is particularly effective for detecting liquid stains, which are often invisible in standard intensity images due to glare or transparency. This dual-modality input serves as the foundation for robust detection in complex environments.

#### 3.2.2. Hierarchical Feature Extraction Backbone

**Figure 3:**
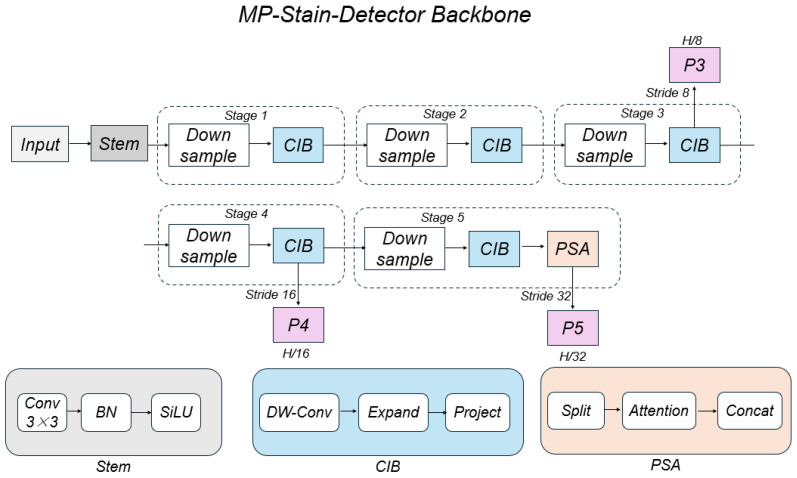
The architecture of the hierarchical feature extraction backbone. It consists of a Stem module for initial processing, followed by five stages containing Downsample layers and Compact Inverted Blocks (CIB). A Partial Self-Attention (PSA) module is integrated at the end of Stage 5 to capture global context. The detailed structures of Stem, CIB, and PSA are illustrated in the bottom panels.

To efficiently extract discriminative features from the high-dimensional input $I_{in}$, we employ a state-of-the-art hierarchical backbone network designed for an optimal trade-off between computational efficiency and representation capability. This backbone is built upon the Cross Stage Partial (CSP) network principle but integrates advanced structural re-parameterization and attention mechanisms to enhance feature extraction. The overall architecture is illustrated in Figure 3.

The backbone processes the input through a series of hierarchical stages. First, a Stem module, comprising a standard 3×3 convolution with Batch Normalization (BN) and SiLU activation, maps the input tensor into the initial feature space and performs preliminary downsampling. For robotic cleaning applications, the detection system must process high-dimensional multispectral data using limited onboard computational resources. Standard convolutions, however, are computationally expensive and susceptible to redundancy when processing spectrally correlated bands. To address this, we employ the Compact Inverted Block (CIB) as the core building unit across multiple stages to achieve an optimal balance between efficiency and feature representation. By leveraging low-rank approximations, the CIB module effectively aggregates local spectral-polarization features while minimizing computational overhead. Unlike standard convolutions, the CIB module utilizes a depthwise separable convolution strategy—comprising a depthwise convolution (DW-Conv), a pointwise expansion (Expand), and a projection layer (Project)—to reduce computational redundancy while preserving the representational capacity essential for describing fine-grained stain textures.

Furthermore, distinguishing complex stains from intricate floor patterns requires global context, as local visual cues are often ambiguous due to environmental noise or specular reflections. Traditional Convolutional Neural Networks (CNNs) are limited by their local receptive fields, hindering their ability to capture the long-range dependencies necessary for identifying large-scale or scattered contaminants. Although standard Multi-head Self-Attention (MSA) mechanisms effectively capture global information, they incur prohibitive computational costs for high-resolution feature maps. To overcome this limitation, a Partial Self-Attention (PSA) module is integrated at the final stage of feature extraction (Stage 5). The PSA module selectively applies self-attention to a subset of feature channels, enhancing the global receptive field with minimal computational cost, thereby significantly improving the model’s ability to differentiate stains from the background.

Mathematically, the backbone transforms the input $I_{in}$ into a set of multi-scale feature maps $F=\{P_{3},P_{4},P_{5}\}$. These feature maps correspond to different downsampling strides of 8, 16, and 32, respectively:

(2) $$\begin{array}{c} P_{3}\in R^{\frac{H}{8}\times \frac{W}{8}\times C_{3}}\\ P_{4}\in R^{\frac{H}{16}\times \frac{W}{16}\times C_{4}}\\ P_{5}\in R^{\frac{H}{32}\times \frac{W}{32}\times C_{5}} \end{array}$$

where $C_{3},C_{4},$ and $C_{5}$ denote the channel dimensions of the feature maps at each scale. The hierarchical design allows the network to capture features at different levels of abstraction. Low-level features ($P_{3}$) are enriched by polarization data, sharpening edges and enhancing texture details, which is crucial for precise boundary localization. High-level features ($P_{5}$) benefit from spectral consistency across bands, enabling the network to learn robust semantic representations of different stain categories. This ensures that the network can simultaneously achieve precise boundary detection and accurate classification.

#### 3.2.3. Multi-Scale Feature Fusion with PANet

Stains in real-world scenarios exhibit extreme scale variations, ranging from small droplets to large liquid spills. To address this challenge, we employ a Path Aggregation Network (PANet) to effectively fuse features across different scales. This architecture augments the standard Feature Pyramid Network (FPN) with an additional bottom-up path, thereby establishing a bidirectional information flow.

First, the top-down pathway propagates strong semantic features from higher levels to lower levels. For instance, $P_{5}$ is upsampled using nearest-neighbor interpolation to match the spatial resolution of $P_{4}$, and is subsequently concatenated with it:

(3) $$F_{4}^{td}=\mathrm{Concat}\left({\mathrm{Upsample}}_{NN}\left(P_{5}\right),P_{4}\right)$$

where ${\mathrm{Upsample}}_{NN}\left(\cdot \right)$ denotes the nearest-neighbor interpolation operation. Subsequently, the bottom-up pathway propagates precise localization signals from lower levels to higher levels. Specifically, a 3×3 convolutional layer with a stride of 2 is employed for downsampling to simultaneously reduce the spatial dimensions and increase the channel depth. The resulting fused feature map at level i+1, denoted as $N_{i+1}$, is computed as follows:

(4) $$N_{i+1}=\mathrm{Concat}\left({\mathrm{Conv}}_{3\times 3,s=2}\left(N_{i}\right),F_{i+1}^{td}\right)$$

Through this process, the network generates a set of enhanced multi-scale features $D=\{N_{3},N_{4},N_{5}\}$. Each feature map $N_{i}$ contains both rich semantic information and precise spatial details, significantly improving the detection performance for both small and large stains. Furthermore, the fusion process integrates spectral and polarization cues across scales, enabling the network to leverage this complementary information for more robust detection.

#### 3.2.4. Decoupled Detection Head and Output

Finally, the fused features are fed into a decoupled detection head. Unlike traditional coupled heads, this approach separates the classification and regression tasks into two parallel branches to mitigate feature conflicts. Furthermore, we adopt a consistent dual assignment strategy during training, which enables the model to perform end-to-end detection during inference without requiring non-maximum suppression (NMS) post-processing.

The network output for each grid cell *j* at scale *i* comprises two primary components, denoted as $o_{i,j}=\left(b_{i,j},c_{i,j}\right)$:

1. Bounding Box Regression ($b_{i,j}$): This vector represents the spatial coordinates of the detected stain. It is defined as $b_{i,j}=\left(x,y,w,h\right)$, where (x,y) denote the center coordinates of the bounding box, and (w,h) represent its width and height. The network predicts the Distribution Focal Loss (DFL) values, which are subsequently decoded into absolute image coordinates.
2. Category Classification ($c_{i,j}$): This vector, $c_{i,j}\in R^{K_{cls}}$, represents the predicted probability scores for the $K_{cls}$ stain categories. In this architecture, there is no separate objectness branch; instead, the maximum value in $c_{i,j}$ serves as the confidence score for the detection, implicitly indicating the presence of a stain.

The final set of detections D is directly obtained by filtering the predictions based on a confidence threshold, thereby ensuring high inference efficiency:

(5) $$D={\left\{\left(b_{k},\mathrm{argmax}\left(c_{k}\right),\max\left(c_{k}\right)\right)|\max\left(c_{k}\right)>\tau_{conf}\right\}}_{k=1}^{M}$$

where *M* denotes the total number of detected stains, $\mathrm{argmax}(c_{k})$ determines the predicted category, and $\max(c_{k})$ represents the final confidence score. This decoupled design is motivated by the observation that classification and localization rely on distinct types of features: classification depends more on spectral signatures (for material identity), while localization relies heavily on polarization-enhanced structural cues (such as edges and shapes). By decoupling these tasks, the network can optimize each objective independently without mutual interference. Additionally, the NMS-free design significantly reduces inference latency, making it highly suitable for real-time robotic applications, as it guarantees that inference speeds outpace the system’s hardware acquisition limits (further detailed in Section 4.4).

#### 3.2.5. Dual-Head Loss Function

To achieve end-to-end detection without non-maximum suppression (NMS), we introduce a consistent dual assignment strategy during training. The network is supervised by two heads: a one-to-many head that provides rich supervisory signals for convergence, and a one-to-one head that ensures a unique prediction for each ground truth instance. The total loss $L_{total}$ is the weighted sum of the losses from both heads:

(6) $$L_{total}=L_{o2m}+\lambda L_{o2o}$$

where $L_{o2m}$ and $L_{o2o}$ represent the losses for the one-to-many and one-to-one branches, respectively, and λ is a balancing coefficient. Both branches employ the same loss formulation, which comprises a classification loss $L_{cls}$ and a bounding box regression loss $L_{box}$:

(7) $$L=\lambda_{cls}L_{cls}+\lambda_{box}L_{box}$$

For the classification task, we utilize the Binary Cross Entropy (BCE) loss to measure the discrepancy between the predicted category probability *p* and the ground truth label *y*:

(8) $$L_{cls}=-\frac{1}{N_{pos}}\sum_{i}\left[y_{i}\log\left(p_{i}\right)+\left(1-y_{i}\right)\log\left(1-p_{i}\right)\right]$$

where $N_{pos}$ denotes the number of positive samples. For the bounding box regression task, we combine the Complete Intersection over Union (CIoU) loss and the Distribution Focal Loss (DFL) to ensure precise localization. The regression loss is defined as:

(9) $$L_{box}=L_{CIoU}+L_{DFL}$$

The CIoU loss accounts for the overlap area, center point distance, and aspect ratio consistency:

(10) $$L_{CIoU}=1-IoU+\frac{\rho^{2}\left(b,b^{gt}\right)}{c^{2}}+\alpha v$$

where IoU represents the intersection over union between the predicted box b and the ground truth box $b^{gt}$, ρ(·) denotes the Euclidean distance between their center points, *c* indicates the diagonal length of the smallest enclosing box, and αv serves as the aspect ratio penalty term. The DFL refines the box boundaries by modeling the regression values as a general distribution:

(11) $$L_{DFL}\left(S_{i},S_{i+1}\right)=-\left(\left(y_{i+1}-y\right)\log\left(S_{i}\right)+\left(y-y_{i}\right)\log\left(S_{i+1}\right)\right)$$

where *y* denotes the continuous target value for a box coordinate, and $S_{i},S_{i+1}$ represent the probabilities of the two nearest discrete values $y_{i},y_{i+1}$ ($y_{i}\leq y\leq y_{i+1}$). The CIoU loss ensures the geometric alignment of the predicted boxes, while the DFL refines the localization of ambiguous boundaries, which are common for liquid stains with soft edges. This composite loss function drives the network to learn discriminative features that are robust to environmental noise, fully leveraging the potential of the multispectral polarization data.

### 3.3. Multispectral Polarization Stain Detection Dataset

Traditional stain detection methods based on RGB images exhibit significant limitations: background colors obscure stain details, while ambient light interference (e.g., reflections and shadows) causes edge blurring and reduces local discriminability, consequently lowering recognition recall rates. In contrast, multispectral polarization imaging fundamentally enhances detection performance: its rich spectral features improve material differentiation, while effectively suppressing background light interference and specular reflections on stain surfaces, thereby increasing recognition accuracy and system stability.

However, high-quality datasets for real-world stain detection remain scarce, particularly multispectral polarization datasets acquired in authentic environments. Existing datasets focus primarily on binary stain detection rather than fine-grained category identification.

**Figure 4:**
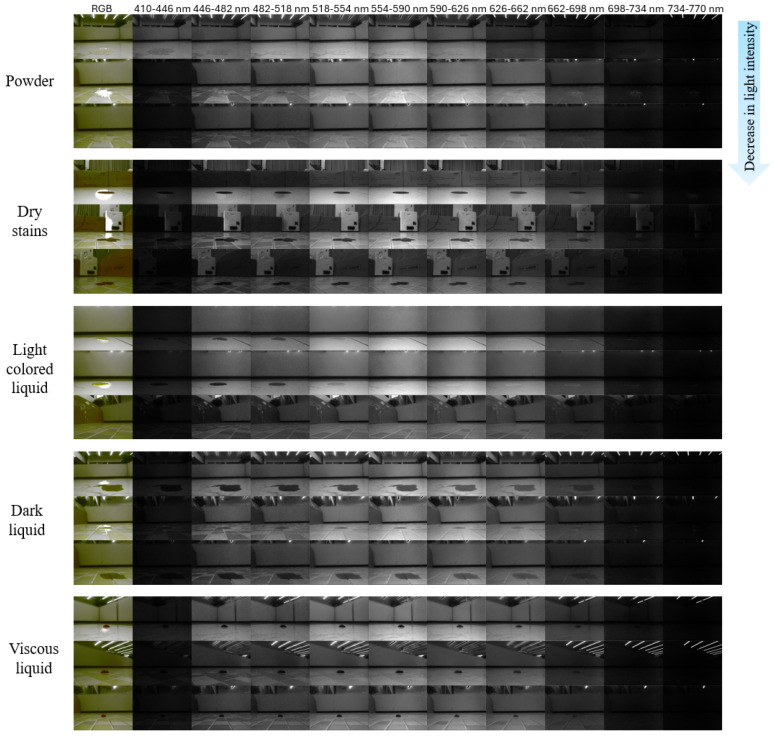
Overview of the MP-Stain-dataset organized by categories and optical modalities. The dataset covers five distinct stain types (columns) captured under varying environmental conditions. For each sample, we provide multidimensional data, including RGB images, multispectral bands (410–770 nm), and specific polarization states (filtered at 45°, 90°, or 135°) to address challenges such as specular reflections and low contrast.

To address this gap, we construct and introduce the MP-Stain-dataset, specifically designed for ground contaminant detection and identification based on multispectral polarization imaging. This dataset classifies common stains into five major categories: dark liquids, light-colored liquids, viscous liquids, dry stains, and powders. As illustrated in Figure 4, we select representative samples from these categories to showcase multispectral images captured under three distinct illumination intensities (strong, medium, and low light) and across different spectral bands, thereby demonstrating the dataset’s robustness to environmental variations. Additionally, we visualize the multispectral optical modalities by presenting the RGB image alongside the ten corresponding multispectral band images. The key features of this dataset include:

- Authentic data provenance: All data were captured using our custom-built multispectral polarization acquisition system (integrated into a cleaning robot platform) within simulated home environments, ensuring scenario authenticity and perspective validity.
- Granular contaminant categorization: Systematically classified into five categories (as detailed above), the dataset provides multispectral images fused with polarization information, capturing optical response characteristics across discrete spectral bands (410–770 nm) combined with polarization states.
- Substantial scale and annotation: The dataset currently comprises 4473 highly annotated multispectral polarization image samples. The data distribution for each stain category is detailed in Table 1, and the dataset remains under active expansion.

This release establishes a critical benchmark for developing and evaluating robust, high-precision multimodal stain detection algorithms, thereby advancing research in this field.

To validate the proposed method, we utilize the MP-Stain-dataset for model training and testing. Simultaneously, we train a model on a corresponding pseudo-color dataset for comparison to verify the superiority of our approach.

## 4. Experiments

### 4.1. Experiment Setup

#### 4.1.1. Hardware Configuration

**Figure 5:**
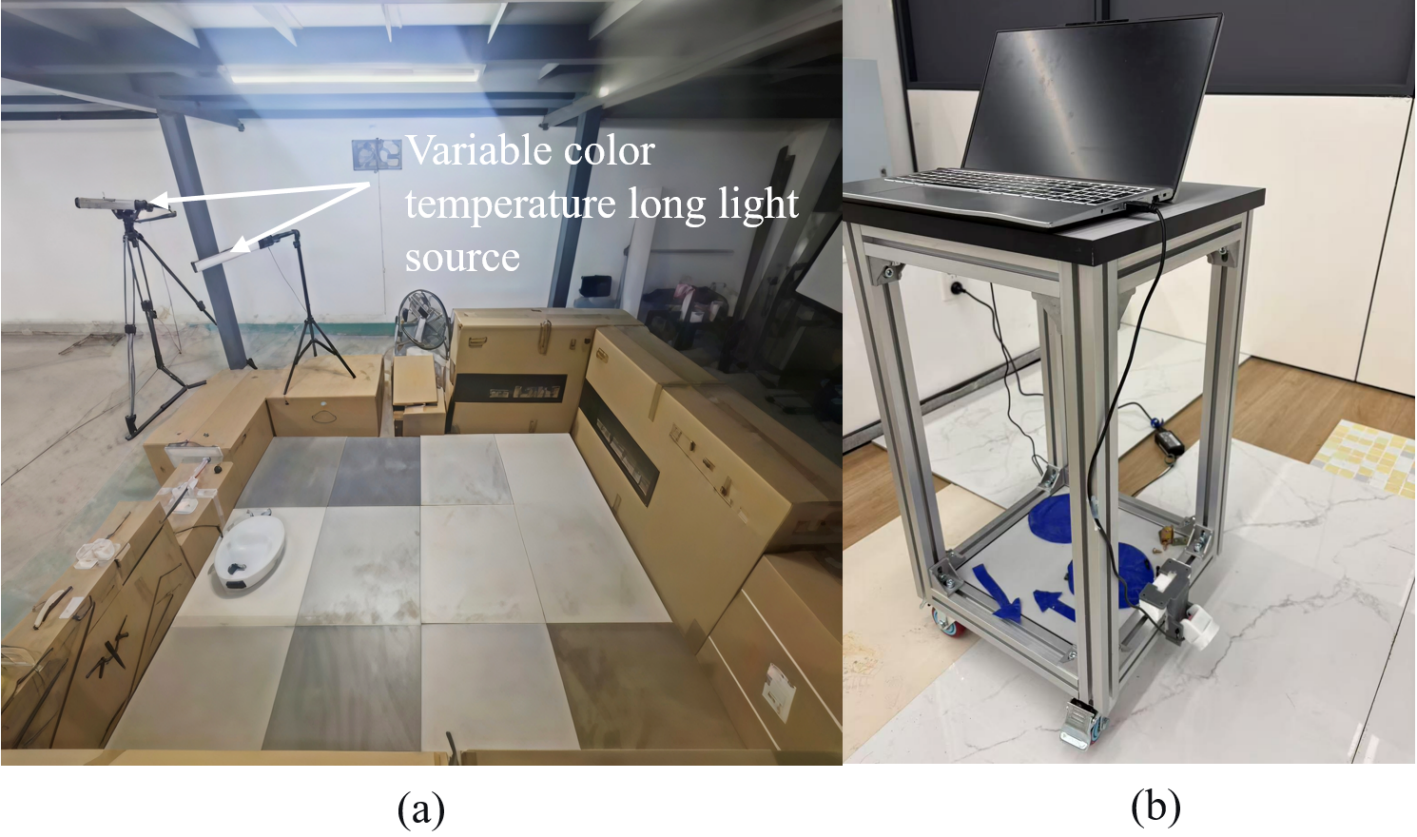
Multispectral polarization imaging system for stain detection. (**a**) The experimental environment for data collection. (**b**) The data acquisition system.

To validate the proposed framework, we constructed a physical prototype of the multispectral polarization imaging system, as shown in Figure 5. The system is equipped with a CM020-series hyperspectral camera, which operates within a spectral range of 350–950 nm. The camera captures images at a resolution of 1600×1200 pixels. Specifically, the camera operates at a maximum hardware frame rate of 6 frames per second (FPS) with an exposure time of 31.2 ms. During operation, the optical center of the camera is positioned at a working distance of 5 cm from the ground, providing an effective Field of View (FOV) of 75.8° (diagonal), 63.54° (horizontal), and 49.8° (vertical).

Regarding the selection basis for the spectral channels (amount, central wavelengths, and widths), these parameters are intrinsically determined by the camera’s hardware calibration and its accompanying data inversion pipeline. Constrained by hardware costs, we selected a specific CM020 variant that operates optimally under indoor LED lighting conditions. During a single capture session, the sensor outputs a proprietary raw data file (a .qs file) along with a broadband grayscale image (utilized in Section 3.2.1). Through the manufacturer’s inversion algorithm, this raw data is strictly decoded into a fixed amount of 10 distinct narrow-band multispectral images. These channels are structurally mapped across the 410–770 nm range with central wavelengths uniformly spaced at intervals of approximately 36 nm. Furthermore, each discrete spectral channel possesses a predefined spectral resolution (Full Width at Half Maximum, FWHM) of 10 nm. This specific hardware-defined configuration provides an ideal balance, capturing sufficient spectral signatures for material discrimination without generating redundant data that would overwhelm the moving robot’s computational resources.

Lighting is provided by two MS-30L variable-color-temperature Light Emitting Diode (LED) (CAMKA COMPANY LIMITED, Taibei, Taiwan, China) strip lights positioned on one side of the capture area. Each light contains 144 LED chips, offering a color temperature range of 3300 K to 5500 K and a high Color Rendering Index (CRI) of Ra > 90. This ensures high color fidelity and allows for the simulation of various lighting conditions, including indoor natural light, strong direct light, and low light. Regarding the specific optical scheme, a polarizing filter assembly is mounted directly onto the front threads of the camera lens to selectively filter the incoming light. The sequentially arranged optical sequence—front-mounted polarizer, focusing lens assembly, and finally the CMOS sensor plane with its on-chip nano-coated spectral filter arrays—ensures that the light is optimally conditioned to remove specular glare before discrete spectral sampling occurs. During data acquisition, the polarizer was set to capture one of the three predefined orientation angles (45°, 90°, or 135°) to obtain optimal samples for specific scenes, effectively eliminating strong specular reflections from liquid contaminants and smooth surfaces.

Regarding system calibration (spectral, spatial, and radiometric), our approach is tailored to the practical constraints of autonomous robotic sweepers. Spectrally, the discrete center wavelengths and bandwidths are strictly locked by the physical nano-coating process on the sensor and factory-calibrated, requiring no further adjustments. Spatially, geometric lens distortions are intrinsically corrected by the manufacturer’s accompanying image inversion software prior to outputting the multispectral images. Crucially, regarding radiometric calibration, conventional multi-band imaging often employs standard test charts (e.g., Spectralon white references or MacBeth ColorCheckers) to convert sensor digital numbers (DNs) into absolute reflectance. However, deploying reference charts is physically unfeasible for dynamically moving robots in unpredictable indoor environments. Furthermore, introducing a front-mounted polarizer naturally alters the absolute incident radiometric intensity. Therefore, specific test charts were deliberately omitted. Instead, as detailed in Section 3.2.1, the proposed end-to-end MP-stain-detector relies directly on the raw spatial-spectral intensities (normalized DNs) captured under the active LED illumination. Deep feature extraction natively learns the discriminative relative intensity distributions rather than absolute radiometric reflectance, demonstrating that the system can operate robustly without complex in-situ radiometric calibration procedures.

#### 4.1.2. Data Collection Process

To ensure high-quality samples for the stain detection algorithm and improve experimental efficiency, we implemented a fully automated stain data acquisition process. This solution integrates diverse experimental variables with precise robotic control to generate a standardized dataset.

During the experimental preparation phase, we focused on diversifying stain categories and environmental conditions. We selected five major categories of stains commonly encountered in daily life: dark liquids (e.g., soy sauce, light soy sauce), light-colored liquids (e.g., iced black tea, Fanta, water), viscous liquids (e.g., oyster sauce, ketchup), dry stains (e.g., dried soy sauce stains, dried light soy sauce stains), and powders (e.g., cat food, cat litter, rice). To further enrich data diversity and improve model generalizability, we arranged various patterned floor tiles in the indoor scene and adjusted the ambient lighting intensity by controlling the brightness of the lamps.

During the acquisition phase, the system first established remote control channels by connecting to the robot’s control system via the Secure Shell (SSH) protocol and to the Human-Machine Interface (HMI) node via the Socket protocol. Following a predefined program, the robot sequentially moved to designated waypoints and precisely aligned itself with each stain point. For each point, the system directed the robot to rotate to the appropriate angle and then acquired images from both the on-board camera and an external camera under three lighting conditions (strong, medium, and low).

**Figure 6:**
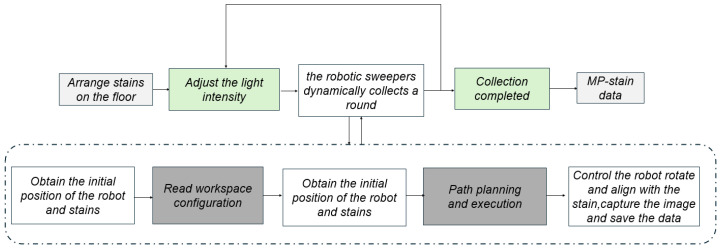
Schematic diagram of the automated data acquisition process.

For data management and monitoring, the collected data were saved in a structured directory format (e.g., stain ID/direction). A real-time visualization interface displayed the robot’s position and the stain distribution, allowing users to initiate the next round of acquisition via the Enter key and trigger emergency stops via the ESC key. Furthermore, a configuration file recorded previously captured stain points to prevent redundant captures, thereby ensuring efficient and complete data collection. The schematic diagram of the acquisition process is illustrated in Figure 6.

### 4.2. Optical System Verification

**Figure 7:**
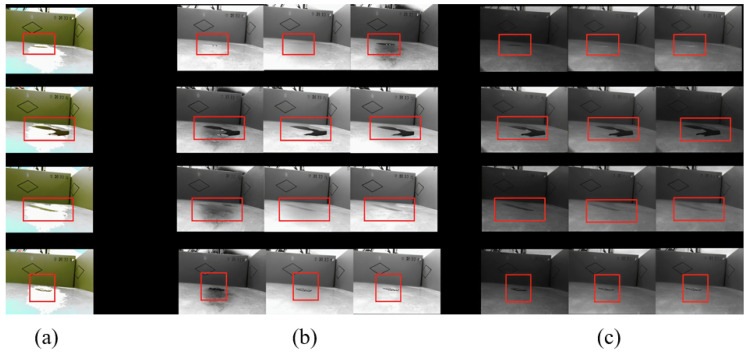
Comparison of RGB, multispectral, and multispectral polarization images. (**a**) RGB: Severe overexposure, reflection, and color interference. (**b**) Multispectral images of some bands: Overexposure shadows, ground light reflections, blurred edges of stains. (**c**) Multi-spectral-polarized image of some bands: Although the brightness is reduced, the stain edge profile is clearer, breaking through the interference of ambient light, background color, and reflected noise. The red boxes highlight specific stain details and specular reflections to facilitate visual comparison across the different optical modalities.

To demonstrate the superiority of the proposed optical system, we conducted a comparative analysis of RGB, multispectral, and multispectral polarization images. Figure 7 presents a visual comparison across different stain types.

As illustrated in Figure 7, RGB images (left column) rely heavily on color contrast. For stains that are similar in color to the background (e.g., light-colored liquids), the contours are blurred, and details are often lost due to specular reflections. Multispectral images (middle column) improve upon this by revealing material differences through reflectance variations across bands. However, they still suffer from specular highlights. The multispectral polarization images (right column) demonstrate the most significant improvement. By filtering out specific polarization states, the system effectively removes specular reflections from the floor and the liquid surface. This results in cleaner backgrounds and sharper stain details, proving that the combination of spectral and polarization information fundamentally enhances the signal-to-noise ratio and feature discriminability.

Multispectral imaging captures optical information across multiple discrete bands from the visible to the near-infrared spectrum, demonstrating significantly richer spectral information compared to traditional RGB imaging for stain detection. Each additional spectral channel reveals the unique reflectance characteristics of the materials, providing a high-dimensional feature space for stain classification. Whereas the three RGB channels primarily convey color and brightness, multispectral data effectively enhance the distinction between stains that are morphologically similar but compositionally different. This multidimensionality also improves detection robustness and strengthens the reliability of identification and classification. By comparing and analyzing stain characteristics in RGB versus multispectral images, we observe that in RGB images, stained areas are visually distinguishable from the background primarily through color differences. However, ambient color may mask stain details, and susceptibility to ambient light (e.g., reflections and shadows) often results in blurred edges or diminished local discriminability. In images across different multispectral bands, the light-dark contrast between the stain and the background exhibits significant variation. In some bands, stains appear dark gray due to light absorption, while the background appears light gray due to reflection. The contrast between the two increases sharply, making the stain outlines more pronounced. Furthermore, multispectral bands are not affected by the color blending inherent in visible light, enabling more precise delineation of the physical boundaries of the stains.

**Figure 8:**
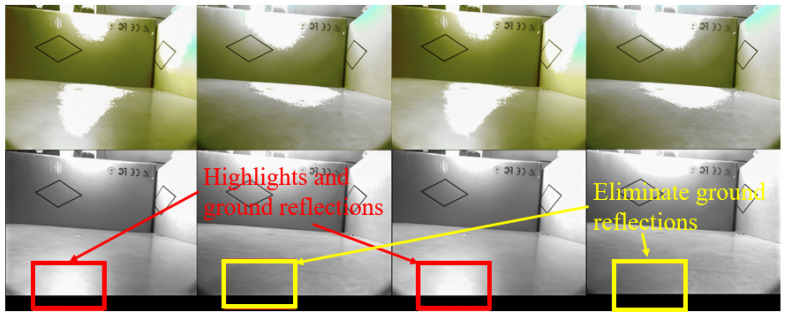
The impact of different polarization angles on the original RGB and grayscale images. An optimal polarization angle (selected from 45°, 90°, or 135°) significantly attenuates ground reflections and eliminates specular highlights, thereby improving imaging quality.

Additionally, leveraging the polarization vector properties of light waves, polarizing filters can physically reduce noise by selectively filtering incident light vibrating in specific orientations. This effectively suppresses specular reflections during imaging. When the polarization angle is adjusted appropriately (e.g., matching the optimal state among the 45°, 90°, or 135° orientations in our dataset), it significantly decreases glare interference, thereby enhancing the image’s signal-to-noise ratio and material texture resolution. Figure 8 shows how the optimal selection of the polarization angle can eliminate glare and improve image quality. For practical robotic deployment, the polarizing filter is fixed at a single angle. However, as the robot continuously moves and rotates across the floor, its relative pose to the ambient light sources changes dynamically. This natural variation in relative orientation ensures that the fixed filter periodically achieves an optimal alignment with the environmental light’s polarization state. Consequently, the moving robot captures high-quality, reflection-suppressed images equivalent to those obtained with an actively adjusted optimal angle, ensuring robust detection without compromising real-time performance.

In summary, from RGB to multispectral imaging and finally to multispectral polarization fused imaging, each added modality enhances image quality while effectively suppressing environmental interference. This progression systematically overcomes limitations imposed by ambient light, background color, and reflection noise, thus expanding the informational dimensions.

**Figure 9:**
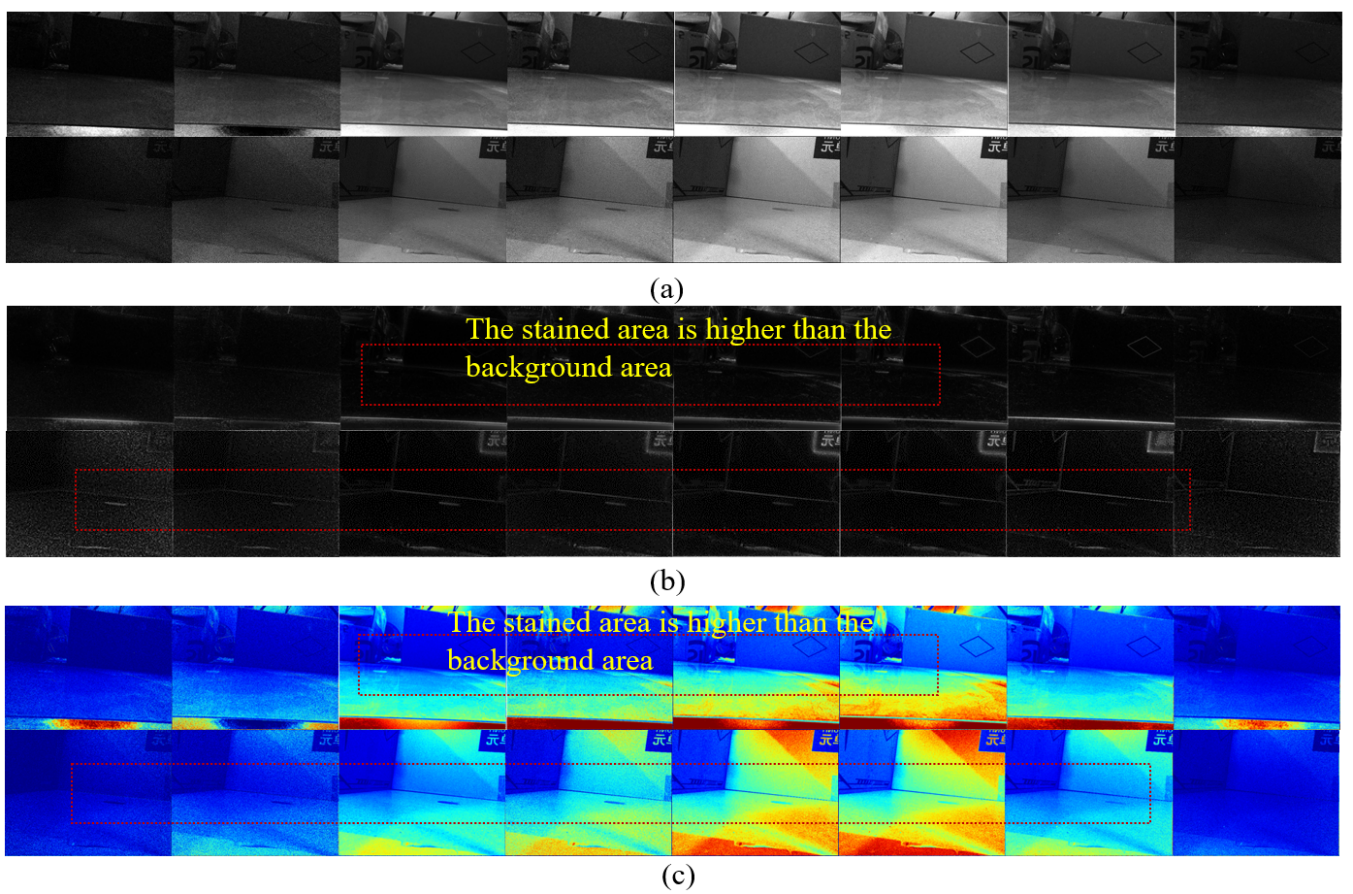
Contrast variations between stains and backgrounds across different spectral bands. (**a**) Original multispectral image. (**b**) Saliency analysis. (**c**) Color space conversion analysis. Compared to RGB images (which have a limited spectrum), multispectral images reveal more abundant salient stain characteristics.

**Figure 10:**
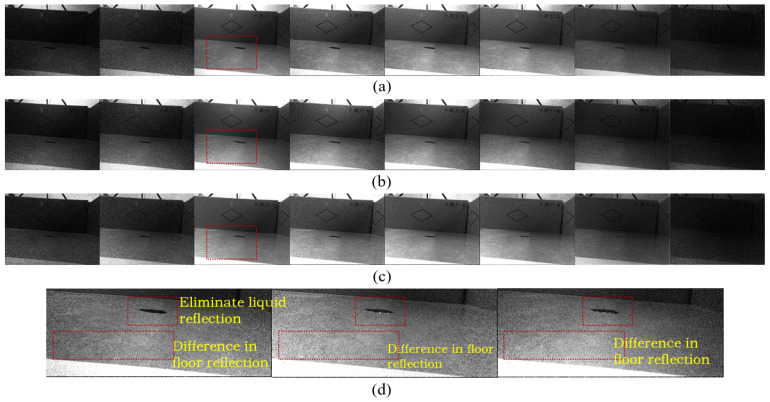
Variations in the elimination of background light interference across different polarization angles (i.e., 45°, 90°, and 135°) in multispectral polarization images. (**a**) Original polarization-multi-spectral image (polarization direction 1). (**b**) Original polarization-multi-spectral image (polarization direction 2). (**c**) Original polarization-multi-spectral image (polarization direction 3). (**d**) Original polarization-multi-spectral image (detail comparison under different polarization directions). The different colors plotted indicate varying intensities of light interference. This variation arises from the complexity of ambient light, where different light sources exhibit distinct polarization angles.

Finally, we conducted a saliency analysis on both the original multispectral images across different bands and the multispectral images incorporating polarization modalities. This analysis identified salient regions within the images, validating the effectiveness of the multispectral polarization acquisition system. As observed in Figure 9, compared to RGB images (which capture limited spectral segments), multispectral images can detect more abundant salient stain characteristics, which is conducive to stain identification and detection, although they are still partially disturbed by ambient light (as are RGB images). As shown in Figure 10a–c, compared to multispectral images, multispectral polarization images can eliminate background light interference, reduce reflections from the stain itself, enhance contour differences between stain and background features, and strengthen salient stain characteristics. Simultaneously, under different polarization angles (specifically 45°, 90°, and 135°), the areas where background light interference is eliminated in the multispectral polarization images differ. This is because the background light is complex, with different light sources exhibiting varying polarization angles, as validated by the results presented in Figure 10d.

### 4.3. Method Verification

To quantitatively evaluate model performance, we employed a series of evaluation metrics, including precision, recall, and mean Average Precision (mAP):

(12) $$\mathrm{Recall}=\frac{TP}{TP+FN}=\frac{TP}{num_{\mathrm{sample}}}$$

(13) $$\mathrm{Precision}=\frac{TP}{TP+FP}=\frac{TP}{num_{\mathrm{pred}}}$$

(14) $$AP=\int_{0}^{1}PR\;dR$$

(15) $$mAP=\frac{1}{C}\sum_{i=1}^{C}AP_{i}$$

where TP denotes true positives, FN denotes false negatives, and $num_{\mathrm{sample}}$ indicates the total number of ground truth objects. FP denotes false positives, and $num_{\mathrm{pred}}$ represents the total number of predicted objects. *C* represents the total number of categories, and $AP_{i}$ represents the Average Precision of class *i*.

**Figure 11:**
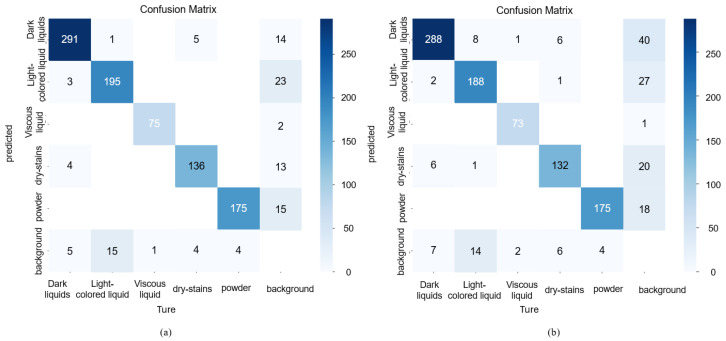
(**a**) Confusion matrix trained on the MP-Stain-dataset. (**b**) Confusion matrix trained on the pseudo-color dataset.

**Figure 12:**
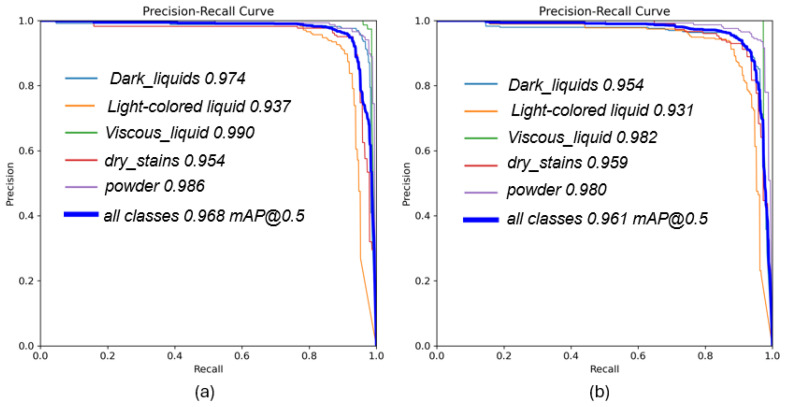
(**a**) Precision–recall curves for validation and test on the MP-Stain-dataset. (**b**) Precision–recall curves for validation and test on the pseudo-color dataset.

Figure 11 presents the confusion matrices for the models trained on the multispectral polarization dataset and the pseudo-color dataset, respectively. The confusion matrix provides a detailed breakdown of classification performance, where diagonal elements represent correct predictions and off-diagonal elements represent misclassifications. As shown in Figure 11a, the model trained on the MP-Stain-dataset exhibits a strong diagonal dominance, indicating high classification accuracy across all categories. In contrast, the pseudo-color model (Figure 11b) shows more dispersion, particularly between ‘Light-colored liquids’ and ‘Background’. This difference highlights the intrinsic advantage of our method: multispectral polarization images simultaneously capture spectral information (reflectance variations across wavelengths) and polarization information (differences in raw intensities influenced by the vibrational direction of light). This enables the extraction of physical properties not available in pseudo-color images, such as the raw polarization intensity variations of transparent liquids and differences in spectral reflectance in powders. Consequently, the MP-stain-detector can robustly distinguish stains that are visually similar to the background in RGB space, directly validating our contribution of enhancing feature discriminability through multidimensional optical data. Figure 12 shows the precision–recall (PR) curves for validation and testing on the MP-Stain-dataset and the pseudo-color dataset.

The mAP@0.5 metric represents the mean average precision across all classes, directly reflecting the model’s comprehensive ability to detect all stains. The curvature and coverage area of the PR curve indicate the model’s ability to maintain precision at varying recall levels. As shown in Figure 12, the PR curve for the multispectral polarization dataset is generally closer to the top-right corner, indicating superior performance. Moreover, in high-recall regions (especially when recall exceeds 0.8), its precision decreases more gradually. This behavior is directly attributed to the stability of the features: multispectral polarization data provide multidimensional information, including spectral and polarization states, enabling more precise discrimination between stains and backgrounds even under challenging conditions. In contrast, the PR curve for the pseudo-color dataset drops more sharply. This is because pseudo-color data rely solely on red, green, and blue color information. For stain categories with colors similar to the background or under varying lighting conditions, the discriminative ability of color features diminishes rapidly, leading to false positives and a drop in precision. This comparison further confirms that our proposed method offers better robustness and generalization capability.

To validate the effectiveness of the proposed method, we first evaluated the performance on a subset of the MP-Stain-dataset (excluding the ‘Viscous liquids’ category). We compared YOLOv11 and YOLOv8 using pseudo-color images derived from the multispectral data. Additionally, we tested a method combining YOLOv10 with the D2-Net keypoint detection algorithm, where keypoints extracted from 10 multispectral bands were reshaped and concatenated with the spectral channels. The results are presented in Table 2. YOLOv11 achieved the highest overall mAP of 0.959. Notably, the D2-Net-based method showed significantly lower performance (0.910), particularly for ‘Light-colored liquids’ (0.768). This can be attributed to the fact that D2-Net relies on local texture and corner features for keypoint description, which are often absent or unstable in transparent liquid stains, leading to poor feature representation.

Subsequently, we conducted extensive comparative experiments against state-of-the-art object detection models on the full MP-Stain-dataset. We compared our MP-stain-detector with YOLOv10 [28], YOLOv11 [26] (trained on pseudo-color images), and a variant of YOLOv11 incorporating a saliency map [29]. Table 3 summarizes the performance (mAP50) of these methods.

The results demonstrate that our MP-stain-detector achieves the highest overall mAP of 0.968, surpassing all comparative models. Specifically, it outperforms the pseudo-color-based YOLOv11 (0.961), indicating that the fusion of multispectral and polarization features provides richer discriminative information than standard pseudo-color data. Notably, in the most challenging ‘Light-colored liquids’ category, our method achieves a mAP of 0.937, a significant improvement over YOLOv11 (0.931) and YOLOv10 (0.900). The lower performance of the comparative methods suggests that standard pseudo-color representations fail to fully capture the subtle physical properties of these stains. Furthermore, the comparison with ‘YOLOv11 + Saliency’ (0.950) reveals that our deep feature fusion strategy is more effective than simply concatenating a saliency map as an additional input channel.

**Figure 13:**
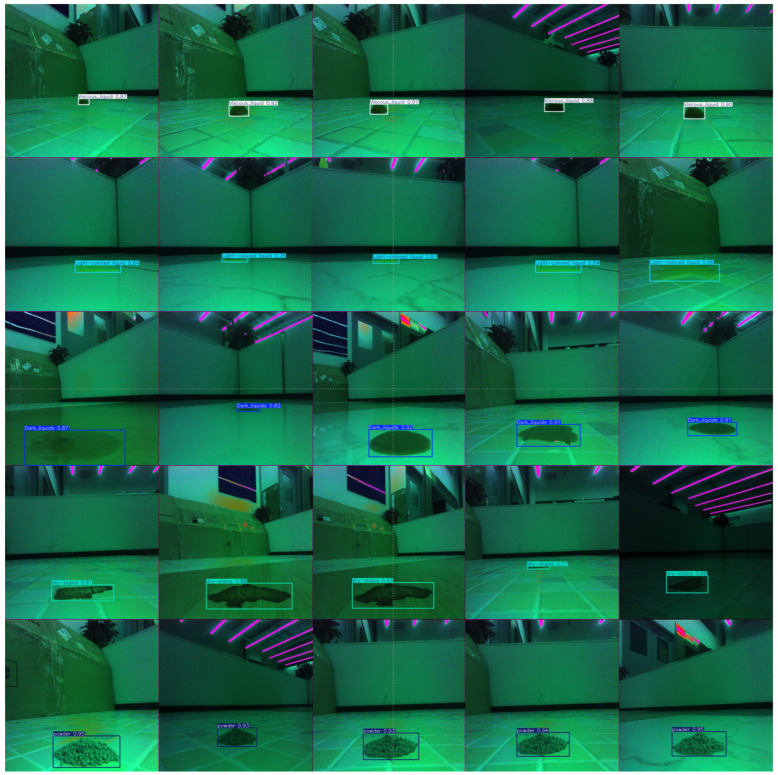
Visualization of model inference detection results. The figure displays the detection performance on the MP-Stain-dataset, including five categories: Viscous liquids, Light-colored liquids, Dark liquids, Dry stains, and Powders. The bounding boxes and class labels indicate accurate localization and classification capabilities.

The qualitative results in Figure 13 visually confirm the quantitative metrics. The model successfully detects stains across all five categories with high confidence scores, as indicated by the bounding boxes. For instance, ‘Viscous liquids’ and ‘Dark liquids’ are accurately localized despite potential similarities in color or texture with the background. Crucially, the ‘Light-colored liquids’ stains, which are typically challenging due to their transparency and low contrast, are also detected with high precision. This visual evidence supports the effectiveness of the multispectral polarization fusion strategy in enhancing feature discriminability for diverse stain materials in real-world scenarios.

### 4.4. Embedded System

To achieve highly efficient stain detection and identification in cleaning robots, we selected the NVIDIA^®^ Jetson Orin^TM^ NX (NVIDIA Corporation, Santa Clara, CA, USA) as the primary computing platform. This series delivers up to 157 Tera Operations Per Second (TOPS) of AI performance within the smallest Jetson form factor, with configurable power consumption between 10 W and 40 W. It delivers 5× the performance of the NVIDIA Jetson AGX Xavier^TM^ (NVIDIA Corporation, Santa Clara, CA, USA) and 7.5× that of the Jetson Xavier^TM^ NX (NVIDIA Corporation, Santa Clara, CA, USA), making it ideal for compact, low-power devices such as robotic sweepers and handheld equipment. Furthermore, to evaluate the real-time applicability of our system, we measured the inference latency on this embedded platform. The proposed MP-stain-detector achieves a processing latency of approximately 73 ms per frame, which translates to an effective frame rate of approximately 13.7 frames per second (FPS). Crucially, because this algorithm inference rate (13.7 FPS) outpaces the maximum physical capture rate of the multispectral camera (6 FPS, as configured in Section 4.1.1), the software introduces no frame buffering or bottleneck, ensuring strict zero-delay synchronization with the sensor’s temporal output. Considering that typical robotic sweepers operate at relatively low speeds (e.g., 0.2 to 0.5 m/s), the robot travels only about 1.5 to 3.6 cm between consecutive frames. This processing speed provides ample reaction time for the control system to adjust its path or deploy targeted cleaning mechanisms before passing over the detected stain, thereby fully satisfying the real-time and in situ performance requirements for autonomous cleaning tasks.

These System-on-Modules (SoMs) integrate an NVIDIA Ampere architecture Graphics Processing Unit (GPU), next-generation deep learning and vision accelerators, high-speed Input/Output (I/O) interfaces, and high memory bandwidth, supporting multiple concurrent AI application pipelines. We accelerated the model inference using TensorRT and compared its performance with an RTX 4090 GPU (NVIDIA Corporation, Santa Clara, CA, USA). Through testing, we observed that TensorRT acceleration on the embedded platform reduced the single-frame inference latency from 171.4 ms to 73 ms, a 57.4% reduction in latency. In contrast, the non-accelerated RTX 4090 GPU exhibited a latency of 123.9 ms, demonstrating that the accelerated embedded system achieves a 40.7% lower latency.

## 5. Discussion

The development of the MP-stain-detector was driven by the inherent limitations of conventional RGB-based vision systems in robotic sweepers. In complex indoor environments, standard cameras frequently struggle with specular reflections and background color interference, leading to missed detections of transparent or camouflaged contaminants. To overcome these critical bottlenecks, we proposed a novel framework integrating a custom multispectral polarization optical system with a specialized deep learning architecture. By capturing multidimensional optical properties, our approach fundamentally shifts the paradigm from relying solely on color contrast to leveraging material-specific spectral signatures and polarization-enhanced textures.

The effectiveness of this proposed solution is comprehensively validated through our experimental results. As demonstrated in Figure 7, the multispectral polarization system successfully eliminates glare and highlights, revealing clear stain boundaries that are otherwise invisible in RGB images. Furthermore, the quantitative evaluations presented in Table 3 confirm that our MP-stain-detector achieves a superior overall mAP of 0.968. Notably, the significant performance improvement in detecting challenging ‘Light-colored liquids’ (achieving an mAP of 0.937) is directly attributable to our core contribution: the deep fusion of spectral and polarization features. The confusion matrices (Figure 11) and PR curves (Figure 12) further corroborate that our multidimensional data representation effectively resolves feature ambiguity, ensuring robust classification even under challenging lighting conditions.

**Scope of Inspected Surfaces and Stains:** The current system is primarily designed for common indoor household environments. During data collection, we arranged various types of flooring based on typical household materials to simulate authentic scenarios. The target stains cover five major categories (dark liquids, light-colored liquids, viscous liquids, dry stains, and powders), which represent the most frequent contaminants encountered by robotic sweepers.

**Data Requirements for Training:** The fusion of multispectral and polarization data inherently increases the feature dimensionality. To support this, our training dataset comprises a total of 4473 annotated images across the aforementioned five categories. This volume of data has proven sufficient to achieve the high detection rates demonstrated in our experimental results, ensuring robust generalization across different lighting conditions.

**Future Directions for System Expansion:** While the proposed system demonstrates exceptional performance in household scenarios, it presents exciting opportunities for further expansion. Currently, the model is highly optimized for the predefined categories and typical indoor flooring. Future research will focus on extending the dataset to encompass more complex industrial surfaces and a broader variety of outdoor contaminants. Additionally, we plan to further optimize the optical module’s size and hardware cost, facilitating its broader commercial deployment across diverse autonomous cleaning platforms.

## 6. Conclusions

In this study, we addressed the challenge of robust stain detection in complex indoor environments by proposing a novel framework that integrates multispectral polarization imaging with deep learning. Our research yielded several key findings. First, we demonstrated that standard RGB imaging is fundamentally limited by its inability to suppress specular reflections and distinguish material properties. By introducing a multispectral polarization optical system, we established that capturing multidimensional optical data significantly enhances stain saliency, particularly for transparent liquids and camouflaged stains. Second, we found that fusing spectral composition data with polarization texture features in a deep learning model (MP-stain-detector) effectively resolves the ambiguity between stains and complex backgrounds. Our experiments on the newly constructed MP-Stain-dataset confirmed that this multimodal approach improves the overall detection accuracy by 2.44% and achieves a remarkable 5.72% improvement for difficult-to-identify light-colored liquids. These results validate that leveraging the physical properties of light—specifically spectrum and polarization—is a viable and superior direction for advancing robotic perception capabilities.

## Figures and Tables

**Table 1:** Data distribution across various stain categories in the MP-Stain-dataset.

Class	All	Dark Liquids	Light-Colored Liquids	Viscous Liquids	Dry Stains	Powders
Num	4473	1345	977	541	709	901

**Table 2:** Comparative experimental results on the dataset subset (mAP50).

Method	Dark Liquids	Light-Colored Liquids	Dry Stains	Powders	All
YOLOv11 [26]	0.983	0.910	0.953	0.988	0.959
YOLOv8 [13]	0.901	0.950	0.944	0.994	0.955
YOLOv10 + D2-Net [27]	0.967	0.768	0.911	0.992	0.910

**Table 3:** Comparative experimental results on the full MP-Stain-dataset (mAP50).

Method	Dark Liquids	Light-Colored Liquids	Viscous Liquids	Dry Stains	Powders	All
YOLOv10 [28]	0.958	0.900	0.980	0.926	0.980	0.949
YOLOv11 (Pseudo-color) [26]	0.954	0.931	0.982	0.959	0.980	0.961
YOLOv11 + Saliency [29]	0.965	0.922	0.990	0.891	0.982	0.950
Ours (MP-stain-detector)	**0.974**	**0.937**	**0.990**	**0.954**	**0.986**	**0.968**

## Data Availability

Data underlying the results presented in this paper are not publicly available at this time but may be obtained from the authors upon reasonable request.
